# A Rare Case of Homicidal Asphyxia Involving Combined Mechanisms By Multiple Assailants

**DOI:** 10.1097/PAF.0000000000001119

**Published:** 2026-02-19

**Authors:** Anna Laura Santunione, Jessika Camatti, Erjon Radheshi, Rossana Cecchi

**Affiliations:** * University of Modena and Reggio Emilia, Modena; † University of Parma, Parma; ‡ Local Health Authority of Reggio Emilia, Reggio Emilia, Italy

**Keywords:** homicide, mechanical asphyxia, smothering, ligature strangulation, traumatic asphyxia, forensic pathology

## Abstract

Mechanical asphyxia is well documented in forensic pathology; however, deaths resulting from the simultaneous action of distinct asphyxial mechanisms remain uncommon and challenging to interpret. We report a case of homicidal asphyxia in a 20-year-old man in whom smothering, ligature strangulation, and traumatic asphyxia acted concurrently. The victim was found inside a locked suitcase. External examination revealed facial cyanosis, subconjunctival and scleral hemorrhages, and patterned cervical abrasions. Autopsy disclosed extensive hemorrhagic infiltration of cervical soft tissues with fracture of the greater horn of the hyoid, blunt-force injuries of the thoraco-abdominal wall with mesenteric hemorrhage, and pulmonary contusions associated with features of acute pulmonary emphysema. Histologic examination confirmed the vitality of the lesions. Toxicological analyses were negative for alcohol, narcotics, psychotropic substances, and common drugs of abuse. Investigative findings, including video surveillance and concordant suspect statements, documented that the victim was restrained and subjected to simultaneous airway occlusion, neck compression, and thoraco-abdominal compression by multiple perpetrators. The integration of pathologic, imaging, and investigative data supported the interpretation of a rapid fatal process in which airway occlusion (smothering), soft-ligature neck compression, and thoraco-abdominal compression acted synergistically to impair ventilation and perfusion, consistent with homicidal mechanical asphyxia. This case highlights the practical challenges of causal attribution when multiple asphyxial mechanisms co-occur and underscores the importance of structured reporting based on multiparametric correlation between pathologic and investigative evidence.

In forensic medicine, *mechanical asphyxia* represents an overarching category encompassing deaths caused by external mechanical forces that critically interfere with ventilation and gas exchange.^1^ Multiple classifications have been proposed to categorize the diverse mechanisms of asphyxia;^2–5^ however, overlaps between categories frequently complicate interpretation in forensic casework.^3^ Deaths may occasionally involve the concurrent action of different asphyxial mechanisms, posing significant challenges in both classification and causal interpretation.^4,6–8^

Homicidal asphyxia is most often attributed to a single perpetrator acting through a single mechanism. More rarely, complex scenarios are encountered in which multiple asphyxial mechanisms act simultaneously, or in which asphyxial and nonasphyxial mechanisms coexist within the same fatal event.^9–17^ Although such cases have been sporadically reported, their recognition and interpretation remain demanding, particularly when autopsy findings must be integrated with investigative evidence to reconstruct the dynamics of the assault.

In particular, the term *burking* derives from the name of William Burke, who, together with his accomplice William Hare, committed a series of murders in Edinburgh in the early 19th century to sell the victims’ bodies to medical schools for anatomic dissection. Their method was specifically designed to induce death rapidly while leaving minimal external signs of violence. From a forensic perspective, burking is classically described as a homicidal form of combined mechanical asphyxia, resulting from the simultaneous obstruction of the external airways and traumatic asphyxia caused by thoracic compression, thereby preventing effective ventilation. In historical accounts, one perpetrator occluded the victim’s mouth and nose, while the other applied force to the chest by sitting or kneeling on it, producing a combination of smothering and traumatic asphyxia.^18^

In modern forensic literature, the definition of burking is not entirely uniform. Some authors emphasize thoracic compression as the defining feature, whereas others describe burking more strictly as the combination of chest compression and manual occlusion of the mouth and nose.^19^ Burking is generally regarded as a particular form of combined homicidal asphyxia, in which multiple mechanical actions act synergistically to impair respiration and circulation. This conceptualization places burking within the broader framework of violent asphyxiation mechanisms that may present with scarce or nonspecific external findings.^20^

Here we present the case of a 20-year-old man who died as a result of a combined homicidal asphyxia involving smothering, ligature strangulation, and thoraco-abdominal compression, highlighting the diagnostic and interpretative challenges posed by the simultaneous action of multiple asphyxial mechanisms.

## CASE-REPORT

### Death Scene Investigation

**FIGURE 1:**
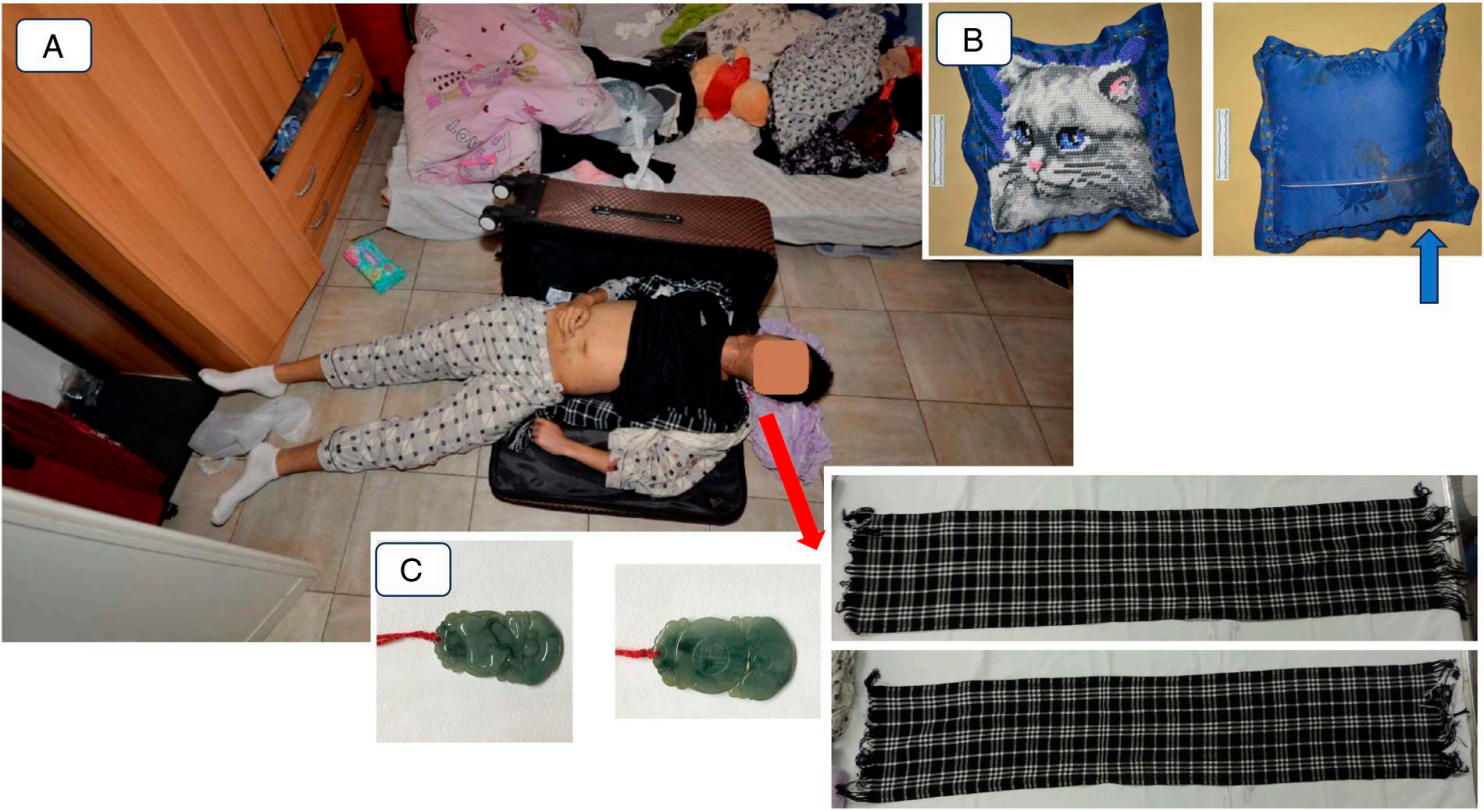
A, Victim at the time of the forensic pathologist’s arrival, after partial removal of the body from the locked suitcase. B, Blood-stained pillow recovered at the scene (blue arrow indicates the bloodstain). C, Pendant necklace observed around the neck. D, Scarf observed around the neck.

The body of a 20-year-old man was discovered inside a locked suitcase in his bedroom. Upon the forensic pathologist’s arrival, the victim was found supine, with his back resting against the interior of the suitcase (Fig. 1A). A blood-stained pillow was recovered at the scene (Fig. 1B). Surveillance footage documented the arrival and departure of 5 acquaintances within 2 hours before discovery.

**FIGURE 2:**
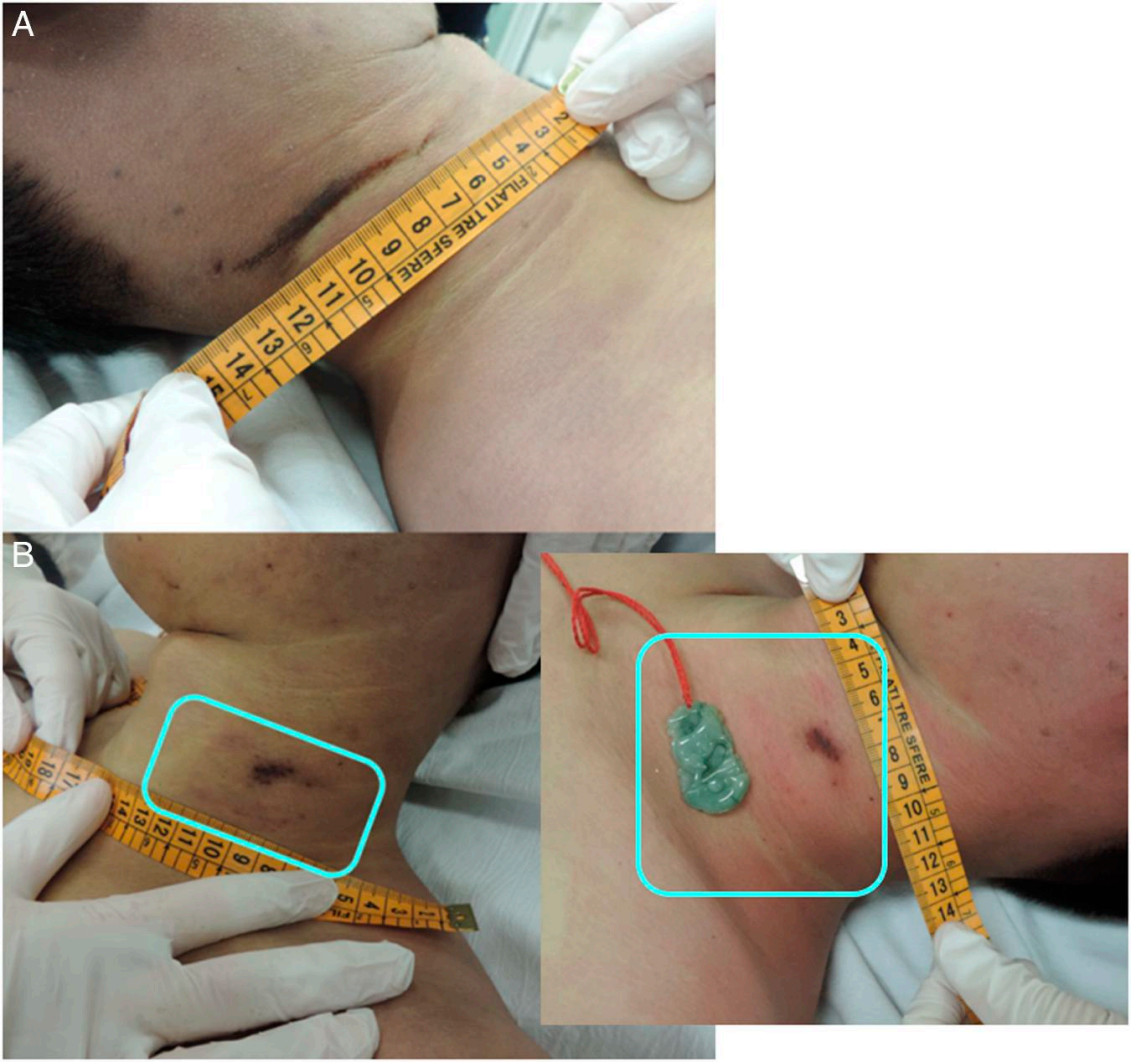
A, Right latero-cervical region, external examination: note a ribbon-like excoriation. B, Left latero-cervical region, external examination: the blue rectangles indicate the patterned abrasion with ecchymosis; please note the pendant.

**FIGURE 3:**
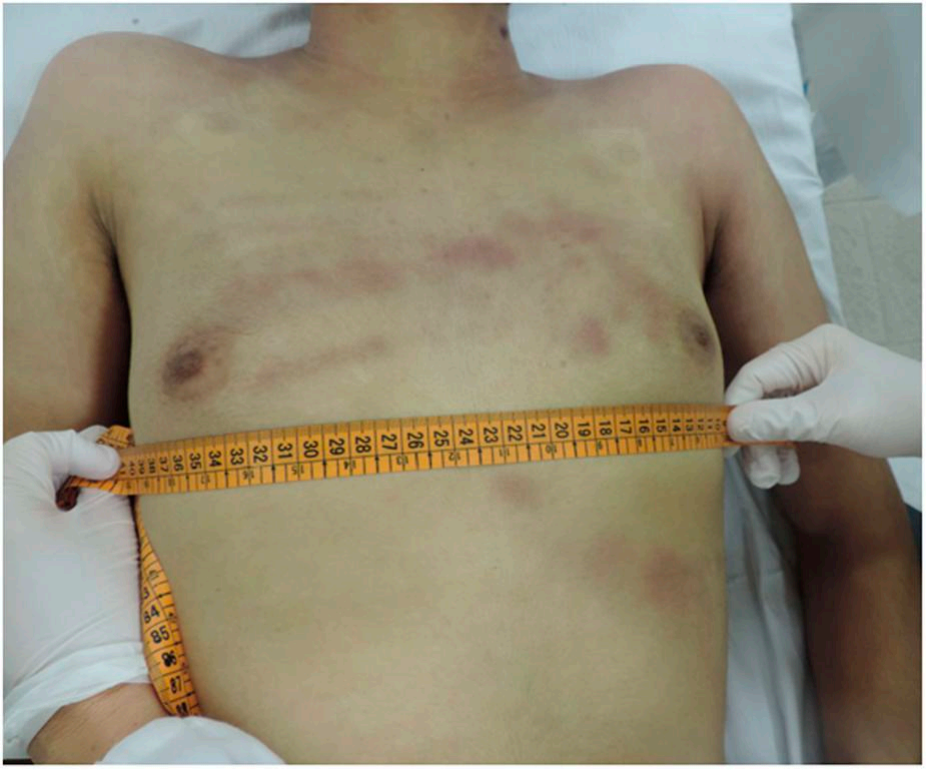
Anterior surface of the chest, external examination: note extensive contusion injury.

At the scene, the forensic pathologist observed facial cyanosis, numerous subconjunctival petechiae, scleral hemorrhages, and petechiae on the vestibular surface of the upper lip. Around the neck were a scarf and a pendant necklace (Figs. 1C, D). External examination disclosed a ribbon-like excoriation (5×0.6 cm) in the right latero-cervical region and a patterned abrasion with ecchymosis (5×3 cm) in the left latero-cervical region (Fig. 2]. Extensive contusions were present over the anterior chest wall (∼26×8 cm) (Fig. 3).

### Forensic Imaging and Autopsy

Before external examination and autopsy, the body underwent postmortem CT with a 3D reconstruction, which revealed a fracture of the greater horn of the hyoid and thickening of the left pectoral muscle.

**FIGURE 4:**
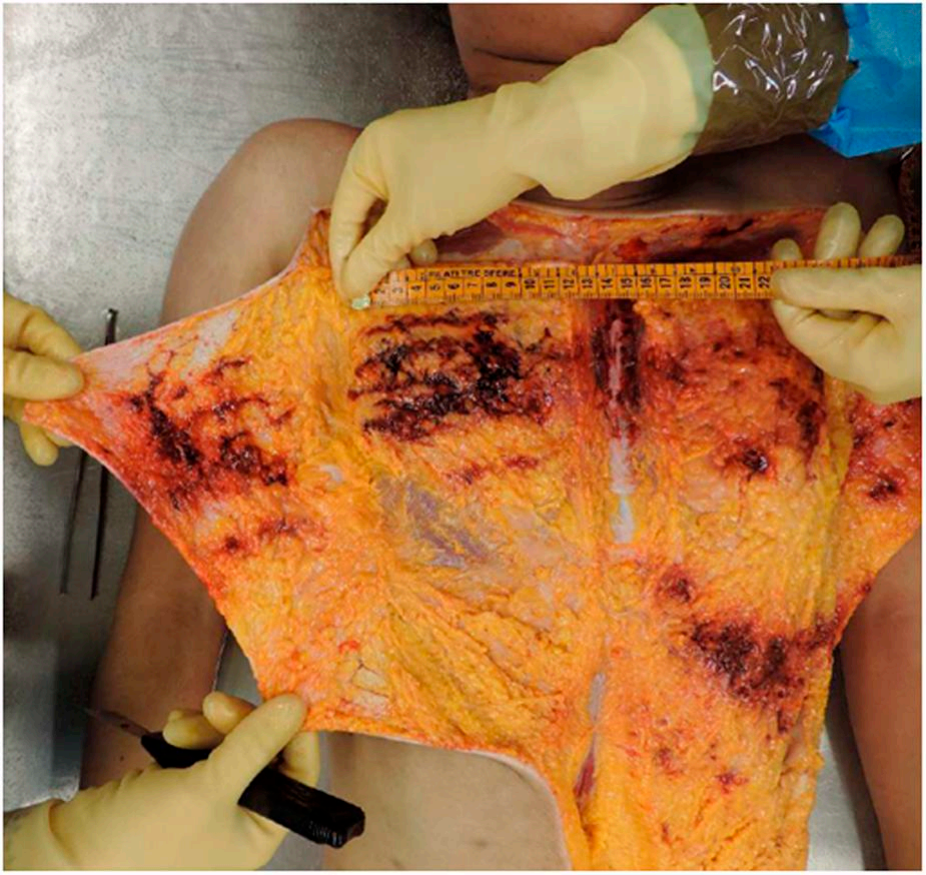
Anterior surface of the chest, autopsy: note large hemorrhages in the subcutaneous soft tissues.

**FIGURE 5:**
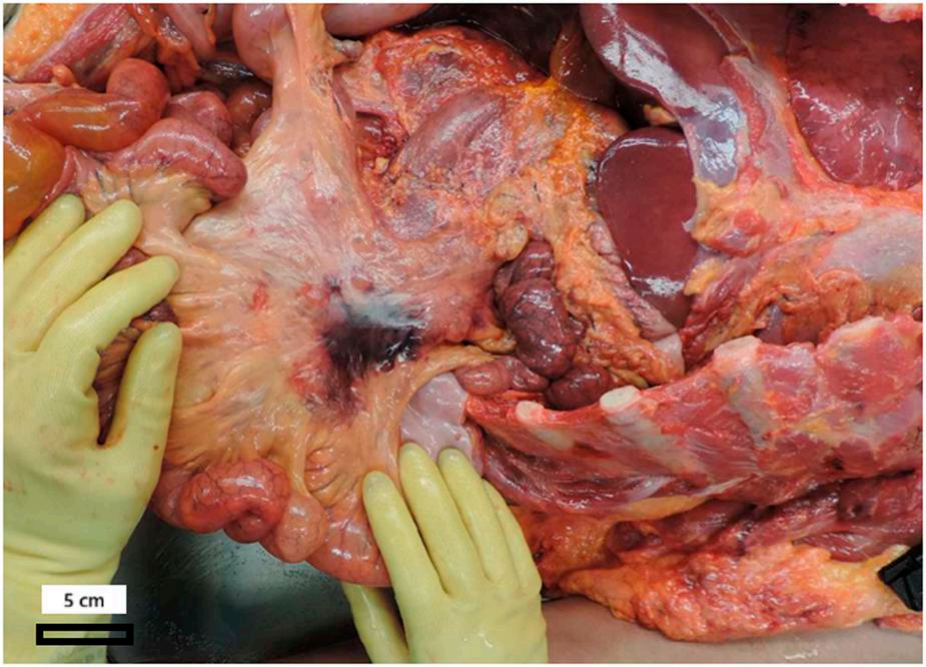
Abdomen, autopsy: note marked hemorrhage in the mesentery.

The decedent was 175 cm tall and weighed 64.5 kg (body mass index ≈ 21.1 kg/m^2^). At autopsy, cerebral edema and congestion (brain weight 1420 g) were noted. Large hemorrhages were documented in the subcutaneous tissues (Fig. 4) and the muscles of the anterior chest wall, with pulmonary contusions (right lung 640 g, left lung 600 g), peri-adventitial hemorrhage of the thoracic aorta, and sub-pleural and sub-epicardial petechiae. Abdominal findings included mesenteric hemorrhage (Fig. 5), contusions of the small intestine at the ligament of Treitz, hepatic contusion near the left triangular ligament, and bilateral hemorrhage of the psoas muscles.

**FIGURE 6:**
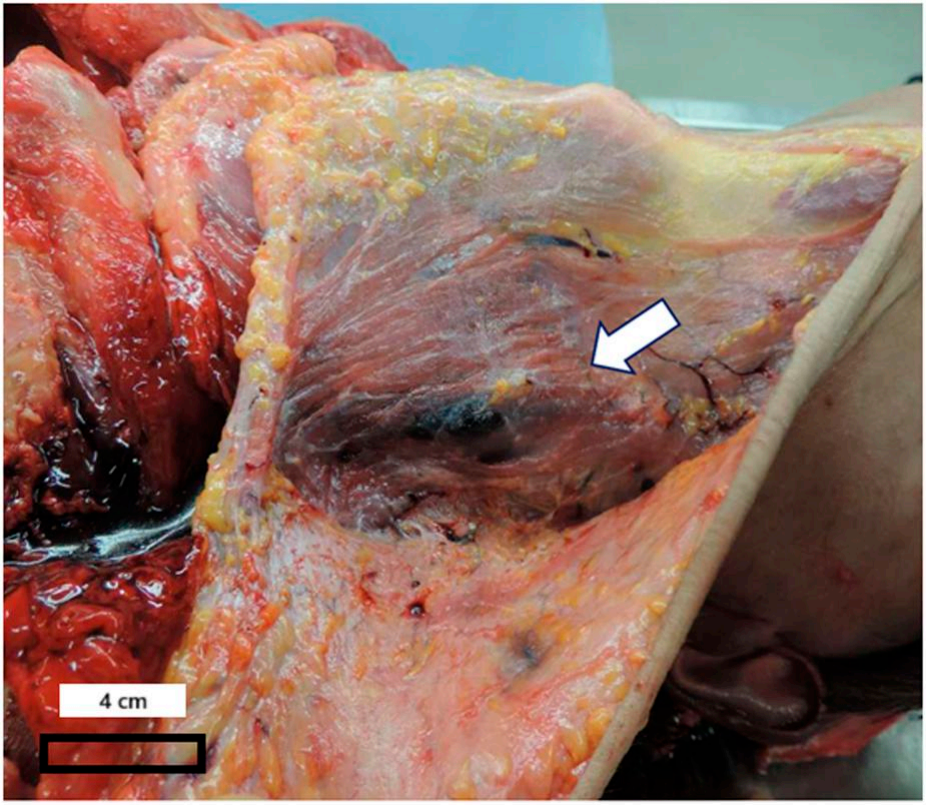
Left latero-cervical region, autopsy: the arrow indicates hemorrhages in the muscular plane.

The cervical region was examined in situ by means of a layer-by-layer dissection. This approach revealed fresh hemorrhage in the soft tissues adjacent to the carotid artery, jugular vein, and vagus nerve sheath, as well as intramuscular hemorrhages of the platysma, sternocleidomastoid, and scalene muscles (Fig. 6). The thyroid gland also showed fresh intraparenchymal hemorrhage. No pre-existing pathologic conditions were identified.

### Histology and Toxicology

Histologic analysis confirmed cerebral edema, vascular congestion, fresh hemorrhages in cervical musculature, and alveolar hemorrhage in contused lung areas. Intrahepatic hemorrhage at the triangular ligament was confirmed.

Toxicological screening of blood, urine, brain, and liver was negative for alcohol, narcotics, psychotropics, and common drugs of abuse.

### Investigation

The victim lived with his mother and her partner, who was present in the apartment during the hours preceding the discovery of the body. He reported that 5 adolescent males had visited the home ∼2 hours before the body was found and had stayed in the victim’s bedroom. One of the boys briefly spoke to him, then left the apartment, stating that the victim had already gone out. The partner subsequently entered the bedroom and found 4 other boys, whom he asked to leave the home. When the victim’s mother returned, she opened a large closed suitcase located in the bedroom and found the body.

Video surveillance footage from the entrance of the building confirmed the arrival and departure sequence of the 5 individuals within the time frame stated by the witness. Their identities were established through telephone records. All 5 suspects—minors at the time of the events—were located and questioned.

Four of them provided concordant statements describing the assault. According to their accounts, the victim was restrained and subjected to airway occlusion using a pillow, neck compression using a scarf placed around the neck, and thoraco-abdominal compression achieved by the perpetrator kneeling on the victim’s chest and sitting on the abdomen, thereby applying their body weight. They further stated that, once death occurred, the body was placed inside the suitcase to conceal it. The fifth individual denied active participation, declaring that he remained outside the room acting as a lookout while speaking to the partner of the victim’s mother.

## Conclusions

The autopsy disclosed the classical external and internal signs of mechanical asphyxia, including facial cyanosis, subconjunctival petechiae, sub-pleural and sub-epicardial petechiae, acute pulmonary emphysema, and generalized visceral congestion. These findings, although nonspecific, supported the diagnosis of death due to mechanical asphyxia.

Three different asphyxial mechanisms were anatomically compatible with the observed injuries: (1) airway obstruction by a soft medium, suggested by the presence of a blood-stained pillow at the scene and oral–nasal frothy fluid without nasal trauma; (2) external cervical compression, supported by patterned abrasions, soft-tissue hemorrhages, and fracture of the greater horn of the hyoid; and (3) thoraco-abdominal compression, indicated by extensive blunt-force injuries of the chest and abdomen, mesenteric hemorrhage, and pulmonary contusions. In particular, the patterned impression on the left latero-cervical region caused by the pendant and the ribbon-like excoriation on the right latero-cervical region caused by the cord suspending the pendant were *suggestive of* compressive forces applied to the neck by a scarf, although these findings are non-specific.

The attribution of these injuries to the simultaneous and coordinated action of multiple perpetrators was consistent with the investigative findings, including surveillance footage and concordant suspect testimony, as detailed in the *Investigation* section.

The convergence of anatomic findings and investigative documentation supports the conclusion that the decedent’s death was caused by the synergistic effects of airway occlusion, ligature-type neck compression, and external thoraco-abdominal pressure, leading to fatal mechanical asphyxia. On the basis of the circumstances established, the manner of death was established as homicide.

## DISCUSSION

The present case documents a homicide in which 3 asphyxial mechanisms—airway obstruction by a soft medium, cervical compression, and thoraco-abdominal compression—were applied simultaneously, producing nonspecific but convergent pathologic findings. The diagnosis of combined mechanical asphyxia was reached only through correlation of autopsy findings, post-mortem CT, scene examination, and investigative evidence, including video surveillance and concordant statements from the perpetrators. The case was further characterized by the concealment of the body inside a suitcase shortly after death. Thus, the present case further reflected the well-recognized diagnostic challenges of asphyxial deaths, which have stimulated continuous research into ancillary markers aimed at supporting medico-legal interpretation.^21–23^

Within this interpretative framework, the dynamics of the assault show partial overlap with mechanisms traditionally described as *burking*.^18–20^ In particular, the combination of airway occlusion and thoraco-abdominal compression reflects a burking-like modality of asphyxiation.

However, the present case cannot be strictly classified as burking. Alongside smothering and thoraco-abdominal compression, ligature strangulation was simultaneously applied, introducing an additional asphyxial mechanism that exceeds the classical burking pattern. The resulting lethal process therefore reflects a complex interaction of concurrent mechanisms, in which no single action can be considered independently sufficient.

In this context, reference to burking is not intended as a diagnostic label but as an interpretative construct, useful for understanding how overlapping asphyxial actions may act synergistically to impair ventilation and circulation. This case thus exemplifies a form of complex combined homicidal asphyxia rather than a classical burking scenario.

Reports of homicidal asphyxia by combined mechanisms show recurring patterns but also relevant differences. Two papers document 3 concurrent modalities (smothering/airway occlusion, neck compression, and thoraco-abdominal/chest compression) perpetrated by a single assailant—in an elderly woman (75 y) and in an adult male—emphasizing typical cervical soft-tissue hemorrhages, hyoid/thyroid fractures, and anterior chest injuries, sometimes with rib fractures.^11,24^ Our case shares the triad of mechanisms and compatible autopsy features, but differs for the young age (20 y), the presence of multiple perpetrators, and the post-event concealment in a suitcase.

In addition to these single-case reports, a small case series further supports the recurring pattern of combined asphyxial actions. A recent case series (n=5) of combined asphyxia (“burking” variants) reported mostly older victims, frequent periorificial injuries, neck/chest muscle hemorrhages, rib fractures, and occasional absence of petechiae, with reconstruction supported by scene context and a single aggressor in all cases.^10^ Our case aligns with the constellation of injuries, but contrasts for age, number of assailants, and investigative documentation (video + concordant statements) directly confirming the simultaneous application of the 3 actions.

In other cases, asphyxial mechanisms have been combined with nonasphyxial methods. For example, Asamura et al^9^ describe a suicide combining suffocation (tape/gag) and ligature strangulation. Although the case described by Asamura and colleagues was a suicide, it illustrates that even when multiple asphyxial mechanisms coexist, the manner of death cannot be determined on autopsy findings alone and requires thorough scene and investigative correlation. The same interpretive limitation applies to multi-mechanism homicidal deaths.^9^

Similarly, Camatti et al^15^ present a femicide with dual noxae (chloroform poisoning plus suffocation) where digital/forensic investigations clarified dynamics and responsibility; although toxicological and criminological contexts differ, the case reiterates the value of explicitly integrating investigative records into the medico-legal synthesis when modalities co-occur.

Beyond dual-mechanism cases, a small number of reports describe 3 distinct asphyxial actions. Indeed, Pramanik^25^ reports the homicide of a 74-year-old man involving 3 asphyxial mechanisms (ligature strangulation, traumatic asphyxia, and plastic-bag smothering). The case shares with ours the coexistence of a triad of asphyxial actions and a compatible autopsy pattern, but differs in the advanced age of the victim, the involvement of a single perpetrator, and the absence of direct investigative confirmation, which in our case included video surveillance and multiple concordant statements, as well as postmortem concealment in a suitcase.

Dual-mechanism cases also offer points of comparison, although with different dynamics. Turillazzi et al^26^ describe a homicide–suicide involving 2 combined asphyxial mechanisms (smothering and ligature strangulation with adhesive tape) in pediatric victims, with marked external lesions and vital histologic changes in the neck. The case is comparable in the coexistence of more than one asphyxial action and the nonspecificity of autopsy signs, but differs in victim age, context (intrafamilial), and the absence of multiple assailants.

The concept of “combined homicide” has been proposed to frame such events more broadly. Amadasi et al^27^ introduce the broader category of “combined homicide,” illustrated by a case involving blunt force trauma, strangulation, and stab wounds, and a review noting the rarity of homicides with 3 distinct methods. The relevance to our case lies in the conceptual framework—that the final reconstruction depends on correlating autopsy, imaging, and investigative data—although the mechanisms in their case are not exclusively asphyxial and the perpetrator was a single subject.

Other cases further illustrate the interpretive complexity posed by multiple lethal actions. Gilani et al^28^ describe a homicide involving 3 different methods (firearm injury, cut-throat wound, and stabbing), emphasizing the need for meticulous scene assessment and systematic autopsy to separate primary from secondary injuries. While not asphyxial, the case illustrates the same medico-legal problem posed by multiple simultaneous lethal actions, but differs from ours because the mechanisms were unrelated to oxygen deprivation, and no concurrent compressive or occlusive forces were involved.

A comparable scenario of multi-mechanism homicidal asphyxia was reported by Taff and Boglioli,^29^ who described the death of a 13-year-old boy assaulted by multiple perpetrators and rendered rapidly unconscious by combined chest and neck compression, followed by the introduction of foreign bodies into the upper airway. As in the present case, the victim exhibited nonspecific asphyxial signs and an absence of defensive injuries, reflecting the overwhelming and coordinated nature of the assault. Although the mechanisms differed—mechanical airway obstruction by pebbles in their case versus smothering and ligature compression in ours—both reports highlight the interpretive difficulty posed by complex asphyxial dynamics and illustrate how multi-assailant attacks may produce overlapping, nondiagnostic patterns of injury that require contextual reconstruction.

Beyond individual cases, recent literature has stressed the central role of scene-based evidence in establishing the manner of death. Santelli et al^30^ showed that the distinction between homicide, suicide, and accident may depend not on autopsy findings alone, but on the structured integration of on-site examination, postmortem imaging, contextual reconstruction, and specialist analyses. This methodological principle is consistent with the approach adopted in the present case, in which the final conclusion could be reached only by correlating pathologic data with investigative evidence. In line with the recommendations of the European Council of Legal and Forensic Medicine (ECFLM), the systematic documentation of the death scene remains an essential component of the forensic workflow, particularly in suspected asphyxial deaths, where anatomic findings are often nonspecific and require contextual interpretation.^31–33^

In summary, this case illustrates the interpretive challenges posed by multi-mechanism homicidal asphyxia, in which several actions act simultaneously to impair ventilation and circulation. Reliable reconstruction requires not only detailed autopsy findings but also correlation with investigative data to correctly establish cause and manner of death and to support judicial assessment.
